# Two-Dimensional Speckle Tracking Echocardiography Detects Subclinical Left Ventricular Systolic Dysfunction among Adult Survivors of Childhood, Adolescent, and Young Adult Cancer

**DOI:** 10.1155/2016/9363951

**Published:** 2016-01-28

**Authors:** Anthony F. Yu, Jayant Raikhelkar, Emily C. Zabor, Emily S. Tonorezos, Chaya S. Moskowitz, Roberto Adsuar, Elton Mara, Kevin Huie, Kevin C. Oeffinger, Richard M. Steingart, Jennifer E. Liu

**Affiliations:** ^1^Department of Medicine, Memorial Sloan Kettering Cancer Center, New York, NY 10065, USA; ^2^Weill Cornell Medical Center, New York, NY 10065, USA; ^3^Department of Epidemiology and Biostatistics, Memorial Sloan Kettering Cancer Center, New York, NY 10065, USA

## Abstract

Two-dimensional speckle tracking echocardiography (2DSTE) provides a sensitive measure of left ventricular (LV) systolic function and may aid in the diagnosis of cardiotoxicity. 2DSTE was performed in a cross-sectional study of 134 patients (mean age: 31.4 ± 8.8 years; 55% male; mean time since diagnosis: 15.4 ± 9.4 years) previously treated with anthracyclines (mean cumulative dose: 320 ± 124 mg/m^2^), with (*n* = 52) or without (*n* = 82) mediastinal radiotherapy. The prevalence of LV systolic dysfunction, defined as fractional shortening < 27%, LV ejection fraction (LVEF) < 55%, and global longitudinal strain (GLS) ≤ 16%, was 5.2%, 6.0%, and 23.1%, respectively. Abnormal GLS was observed in 24 (18%) patients despite a normal LVEF. Indices of LV systolic function were similar regardless of anthracycline dose. However, GLS was worse (18.0 versus 19.0, *p* = 0.003) and prevalence of abnormal GLS was higher (36.5% versus 14.6%, *p* = 0.004) in patients treated with mediastinal radiotherapy. Mediastinal radiotherapy was associated with reduced GLS (*p* = 0.040) after adjusting for sex, age, and cumulative anthracycline dose. In adult survivors of childhood, adolescent, and young adult cancer, 2DSTE frequently detects LV systolic dysfunction despite a normal LVEF and may be useful for the long-term cardiac surveillance of adult cancer survivors.

## 1. Introduction

Strategies in cancer diagnostics and therapeutics have improved dramatically over the last several decades, leading to a growing number of adult survivors of childhood, adolescent, and young adult cancer. The 5-year survival rate for all cancers diagnosed before age of 45 exceeds 80% [1]. As of 2008, there were 619,000 survivors of cancer under the age of 40 years [2]. Despite this progress, the long-term noncancer mortality rate in this population remains substantially higher than age- and gender-matched controls [3], with cardiovascular disease as one of the leading causes of death behind disease recurrence and secondary malignancy [4, 5]. Survivors of childhood (age of 0–14 years) and adolescent (age of 15–19 years) cancer are 7 times more likely to die from cardiac-related events compared to age- and sex- specific rates in the US population [3, 6] and 15.1 times more likely than their siblings to develop heart failure [7]. Less is known about the cardiac outcomes in survivors of young adult (age of 20–39 years) cancer.

Late cardiac effects due to anthracycline chemotherapy and mediastinal radiotherapy in adult cancer survivors are well recognized [8], and subclinical changes in left ventricular (LV) structure and/or function may be seen in more than half of survivors exposed to cardiotoxic therapy [9–11]. Anthracycline chemotherapy can lead to dilated cardiomyopathy and heart failure [12]. Mediastinal radiotherapy is associated with heart failure, premature coronary artery disease, pericardial injury, arrhythmias, and valvular abnormalities [13, 14]. Determination of left ventricular ejection fraction (LVEF) by two-dimensional (2D) transthoracic echocardiography is the primary modality used to screen for LV systolic dysfunction. The Children's Oncology Group (COG) and the National Comprehensive Cancer Network (NCCN) recommend screening echocardiography for all survivors of childhood, adolescent, and young adult cancer, with a frequency based on age at first cardiotoxic treatment, exposure to cardiac-directed radiotherapy, and cumulative anthracycline dose [15, 16]. However, due to the inherent interobserver and intraobserver variability of LVEF assessment by echocardiography, a change in LVEF of 9–11% is the minimum that can be recognized with 95% confidence [17]. In addition, a decline in LVEF is considered to be a late finding of cardiotoxicity [18, 19].

Two-dimensional speckle tracking echocardiography (2DSTE) is a novel method that has potential advantages over LVEF or FS measurement in the early detection of LV systolic dysfunction among both children [20, 21] and adults [22, 23] treated with cardiotoxic therapies and is recommended by the American Society of Echocardiography for the evaluation of patients during and after cancer therapy [24]. However, less is known regarding the use of 2DSTE for the detection of LV systolic dysfunction among adult survivors of childhood, adolescent, and young adult cancer. We performed a single institution cross-sectional study to investigate the use of 2DSTE to detect LV systolic dysfunction among adult survivors of childhood, adolescent, and young adult cancer treated with prior anthracycline chemotherapy, with or without mediastinal radiotherapy.

## 2. Methods

### 2.1. Study Population

A total of 134 consecutively referred adult survivors of childhood, adolescent, and young adult cancer followed up in the Adult Long-Term Follow-Up Program at Memorial Sloan Kettering Cancer Center, a program for high-risk cancer survivors, were included in this study. Screening echocardiograms were performed between July 1, 2010, and December 31, 2012. As per COG and NCCN recommendations, screening echocardiograms were performed annually or biennially [15, 16]. All patients were previously treated with anthracycline chemotherapy, with or without mediastinal radiotherapy, and did not have any known history of symptomatic heart failure. The following data was extracted retrospectively from medical records for each patient: age at diagnosis, cancer diagnosis, date of diagnosis, cardiac risk factors (hypertension, diabetes mellitus, and dyslipidemia), cumulative anthracycline dose, total mediastinal radiotherapy dose, and use of cardiac medications (including aspirin, beta blockers, angiotensin converting enzyme (ACE) inhibitors, angiotensin receptor blockers, or statins). As previously described [15], the cumulative isotoxic anthracycline dose was calculated as the sum of the following: doxorubicin + (daunorubicin × 0.833) + (epirubicin × 0.57) + (idarubicin × 5) + (mitoxantrone × 4). Mediastinal radiotherapy was defined as any form of radiotherapy in which the myocardium was within the prespecified radiation field.

### 2.2. LV Function by 2D Echocardiography

Conventional 2D and Doppler echocardiography was performed using a commercially available standard ultrasound scanner (Vivid E9, General Electric Medical Systems, Milwaukee, WI), according to the standardized American Society Echocardiography (ASE) protocol [25]. LVEF was calculated using the modified Simpson's method. Fractional shortening (FS) was calculated using the standard equation. Abnormal LV systolic function was defined as LVEF < 55% or FS < 27%. Mitral inflow velocity pattern was recorded from the apical 4-chamber view with the pulsed-wave Doppler sample volume positioned at the tips of the leaflets during diastole. Peak early (*E*-wave) and late (*A*-wave) diastolic filling velocities were measured and their ratio (mitral *E*/*A*) was calculated. Doppler tissue imaging of the mitral annulus was performed with measurement of the early (*e*′) diastolic velocity at the septal annulus. Diastolic function assessment was based on mitral *E*/*A* ratio and tissue Doppler (*e*′) velocity only.

### 2.3. 2D Speckle Tracking Echocardiography

Three apical views were used to measure peak systolic global longitudinal strain (GLS) and strain rate using dedicated automated software (EchoPAC 12.0, GE Healthcare, Milwaukee, WI). Three points were manually placed at the endocardial border (one in the apex and two at the mitral valve annulus) in each of the apical views, allowing the software to automatically track myocardial movement throughout the cardiac cycle. After careful inspection, manual correction was performed if the automatic detection was suboptimal. Each view was divided into 6 segments, for a total of 18 segments representing the entire left ventricle. In the case of unsatisfactory tracking due to inadequate image quality, that segment was eliminated from analysis. The timing of aortic valve closure in the apical 3-chamber view was used to define end-systole. Longitudinal strain and strain rate curves were generated for each segment. Strain data were expressed in absolute values (|%|). Global radial (GRS) and circumferential (GCS) strain were calculated by averaging the peak systolic strain values in all 6 segments of the parasternal short-axis view at the papillary muscle. Abnormal LV systolic function was defined as GLS ≤ |16|%. This is a conservative estimate of the lower limit of normal that was derived from studies performed in several healthy cohorts using the mean GLS minus two standard deviations [23, 26, 27].

Interobserver variability was assessed by comparing the original GLS, GRS, and GCS calculation with that calculated by a blinded second observer in 20 randomly selected patients. Intraobserver variability was calculated by repeated measurements in 20 patients by the primary reviewer 3 weeks after the initial measurement.

### 2.4. Statistical Analysis

Continuous measures were summarized as median and interquartile range (IQR) whereas categorical measures were summarized as frequency and percent. Patients were stratified based on cumulative anthracycline dose (<250, 250–399, and ≥400 mg/m^2^) and by mediastinal radiotherapy (yes versus no) for the comparison of the 2D echocardiographic and strain indices, and by-group comparisons were tested using Fisher's exact test when categorical and Kruskal-Wallis test when continuous. The prevalence of abnormal GLS and abnormal FS and LVEF was compared using an exact McNemar's test. Dependent variables considered in multivariable linear regression analysis included FS, LVEF, LV mass/BSA, GLS, mitral *E*/*A* ratio, and septal *e*′ velocity. GLS ≤ |16|% was considered the dependent variable in a multivariable logistic regression model. There were too few events to consider FS < 27% or EF < 55% as outcomes in multivariable analysis. Each multivariable regression model incorporated sex, age at echocardiogram, mediastinal radiotherapy (yes or no), and cumulative anthracycline dose (continuous) as independent variables. Interobserver and intraobserver variability were assessed using the intraclass correlation coefficient (ICC). Bland-Altman plots were constructed by plotting the average of the two readings on the *x*-axis versus the difference between the two readings on the *y*-axis. The mean and standard deviation of the differences were calculated. Because of the small sample size, the *t*-distribution was used as the reference distribution to calculate the 95% limits of agreement. A *p* value of less than 0.05 was considered statistically significant. All statistical analyses were performed using either SAS software version 9.2 (SAS Institute, Cary, NC) or R software version 2.13.1 (R Core Development Team, Vienna, Austria).

## 3. Results

### 3.1. Patient Characteristics

A total of 134 survivors of childhood, adolescent, and young adult cancer were included in this study. Demographic and treatment characteristics are provided in Table 1. The median age at echocardiographic follow-up was 31 years (range, 18 to 62 years), and the median interval since diagnosis was 15 years (range, 2 to 39 years). Sarcoma (*n* = 54), Hodgkin lymphoma (*n* = 29), and acute leukemia (*n* = 31) were the most common diagnoses. All patients were treated with anthracycline chemotherapy, and the median cumulative anthracycline dose was 300 mg/m^2^ (range, 27 to 660 mg/m^2^). Less than half of patients (39%) received mediastinal radiotherapy as part of their cancer treatment, with a median dose of 35 Gy (range, 2 to 56 Gy). The overall prevalence of cardiac risk factors such as hypertension and diabetes was low (9.0% and 5.2%, resp.). Patients who received mediastinal radiotherapy as part of their cancer treatment regimen (*n* = 52) had a lower cumulative anthracycline dose exposure (median dose, 279 versus 375 mg/m^2^, *p* < 0.001) compared to those who were treated with anthracycline chemotherapy alone.

### 3.2. LV Structure and Function

**Figure 1:**
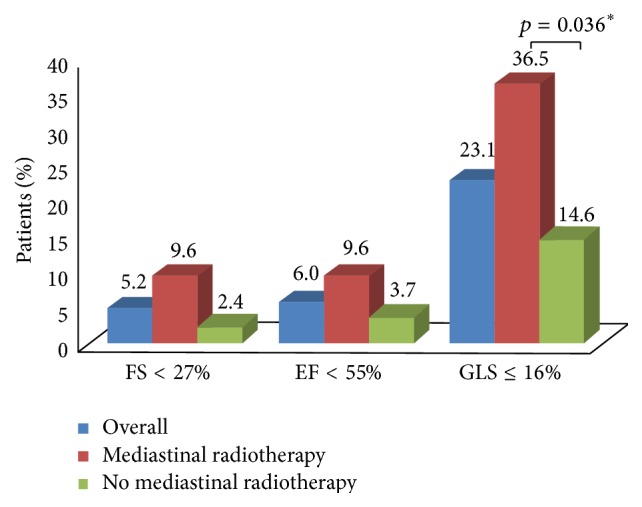
Prevalence of left ventricular systolic dysfunction as measured by left ventricular ejection fraction (LVEF), fractional shortening (FS), and global longitudinal strain (GLS), with or without mediastinal radiotherapy. ^**∗**^ *p* value from multivariable linear or logistic regression adjusted for continuous cumulative anthracycline dose, sex, and age at echocardiogram.

Overall, the mean FS, LVEF, and GLS were 33.3% (range, 23.8% to 44.1%), 61.1% (range, 46.9% to 75.6%), and 18.0% (range, 12% to 26%). LV systolic dysfunction was detected by FS < 27% in 5.2% and LVEF < 55% in 6.0%. However, the prevalence of abnormal GLS ≤ |16|% was 23.1%, significantly higher than abnormal FS (*p* < 0.001) or LVEF (*p* < 0.001) (Figure 1). In the 31 patients with abnormal GLS, only 7 had an abnormal LVEF. There was no significant association between echocardiographic parameters of LV systolic or diastolic function (by 2D echocardiography or 2DSTE) and cumulative anthracycline dose (data not shown).

Echocardiographic parameters of LV systolic function, stratified by mediastinal radiotherapy exposure, are shown in Table 2. GLS was worse in the mediastinal radiotherapy group as compared to the nonradiotherapy group (18% versus 19%, *p* = 0.003) and the prevalence of patients with GLS ≤ |16|% was more than two times greater in the radiotherapy group (36.5% versus 14.6%, *p* = 0.004). However, there was no difference in FS (33.3% versus 33.1%, *p* = 0.686) or LVEF (60.7 versus 61.2%, *p* = 0.301) between the groups with and without mediastinal radiotherapy. Although GCS for the whole study group was significantly lower than the normative reference value (mean 23.3%; 95% confidence interval [CI] 22.1% to 24.6%) based on a recent meta-analysis of normal ranges [28], there was no difference in GCS (16.1% versus 17.6%, *p* = 0.203) or GRS (42.1% versus 42.0%, *p* = 0.843) between the groups with and without mediastinal radiotherapy.

In multivariable linear regression analysis, mediastinal radiotherapy was associated with reduced GLS (beta = 0.923, standard error = 0.444, *p* = 0.040) after adjusting for sex, age at echocardiogram, and cumulative anthracycline dose. Mediastinal radiotherapy was also associated with decreased indices of diastolic function, including lower transmitral *E*/*A* ratio (beta = −0.250, standard error = 0.087; *p* = 0.005) and medial septal tissue Doppler *e*′ velocity (beta = −1.221, standard error = 0.404; *p* = 0.003). There was a trend towards increased LV mass index with higher doses of anthracycline chemotherapy (beta = 0.702, standard error = 0.404; *p* = 0.085) and decreased LV mass index with mediastinal radiotherapy (beta = −3.459, standard error = 2.085; *p* = 0.099), but neither association reached statistical significance.

### 3.3. Intraobserver and Interobserver Variability

**Figure 2:**
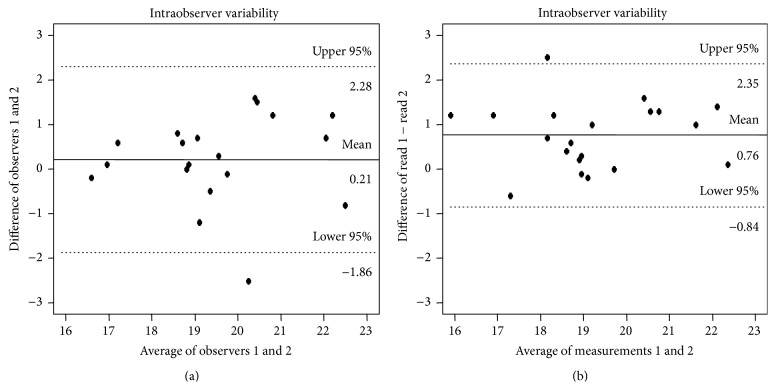
Bland-Altman analysis for interobserver (a) and intraobserver variability (b) for global longitudinal strain measurements in 20 randomly selected patients.

For GLS, the ICC for interobserver agreement was 0.837 (95% CI 0.639 to 0.932) and the ICC for intraobserver agreement was 0.822 (95% CI 0.610 to 0.925), reflecting substantial agreement for measurement of GLS. The Bland-Altman plots indicate that, for interobserver agreement, we expect 95% of measurements from two different readers to differ by between −1.86% and 2.28% whereas, for intraobserver agreement, we expect 95% of measurements taken by the same reader at two times to differ by between −0.84% and 2.35% (Figure 2). For GCS, the ICC for interobserver agreement was 0.74 (95% CI 0.45 to 0.89) and the ICC for intraobserver agreement was 0.93 (95% CI 0.82 to 0.97). For GRS, the ICC for interobserver agreement was 0.83 (95% CI 0.63 to 0.93) and the ICC for intraobserver agreement was 0.77 (95% CI 0.51 to 0.90).

## 4. Discussion

The principle finding of this study is that a significant proportion of adult survivors of childhood, adolescent, and young adult cancer have abnormal LV systolic function detected by 2DSTE despite having a normal LVEF. While an abnormal GLS (≤|16|%) was observed in nearly one-quarter of study participants, a corresponding reduction in FS of <27% or LVEF of <55% was observed in only 5.2% and 6.0% of participants, respectively. These observations suggest that the prevalence of LV systolic dysfunction among long-term cancer survivors may be significantly underestimated using LVEF alone as compared to GLS by 2DSTE.

In the current study, there was no association between cumulative anthracycline dose and conventional or strain indices of LV systolic function. However, this may be attributable to the heterogeneity of the study population and the limited sample size. Indices of diastolic function were significantly lower among patients treated with mediastinal radiotherapy (median dose 35 Gy). This is consistent with a study by Heidenreich et al. in which a high prevalence of diastolic dysfunction was observed among Hodgkin lymphoma survivors treated with at least 35 Gy of mediastinal radiotherapy [29]. Interestingly, the presence of diastolic dysfunction was associated with a poorer cardiac event-free survival.

Although LVEF by 2D echocardiography is the current standard method for monitoring LV systolic function in cancer survivors, myocardial strain by 2DSTE has several important advantages as a screening tool for cardiotoxicity. First, myocardial strain can identify subclinical LV systolic dysfunction prior to a decrement of LVEF. Poterucha et al. found that a reduction in GLS preceded a decrease in LVEF among adolescents receiving anthracycline chemotherapy [21]. Similar results were reported in a study of women with HER2-positive breast cancer in which GLS < |19 | % was predictive of subsequent cardiotoxicity and decreased LVEF [30]. In addition to being a more sensitive marker of LV systolic function, GLS has also been shown to be a predictor of all-cause mortality that may be superior to LVEF or wall motion score index [31]. And, consistent with previous reports, the current study shows excellent reproducibility for measures of peak GLS, as represented by interobserver and intraobserver ICCs of ≥0.80.

Findings from several prior studies support the utility of myocardial strain assessment in cancer survivors [32–35]. Cheung et al. demonstrated that GLS was reduced among asymptomatic children previously treated for acute lymphoblastic leukemia with anthracycline chemotherapy despite having normal fractional shortening [20]. Tsai et al. also showed that GLS was lower among long-term survivors of Hodgkin lymphoma after treatment with anthracycline chemotherapy and mediastinal radiotherapy compared to mediastinal radiotherapy alone [36]. In the current study, GLS was significantly lower and the prevalence of LV systolic dysfunction by 2DSTE was more than two times greater among patients who received both mediastinal radiotherapy and anthracycline treatment compared to those with anthracycline treatment alone. This is consistent with the general finding that longitudinal LV mechanics, which are primarily governed by the subendocardial layer, are the most sensitive and vulnerable to myocardial disease. The circumferential function, which is predominantly governed by the midwall and subepicardial regions, may vary depending on the severity of myocardial involvement. The lack of difference in circumferential strain between the groups with and without radiotherapy may be contributed by the lower reproducibility and accuracy of the circumferential strain measurements.

Less than 10% of the patients were treated with cardioprotective medications, which may confound the association between mediastinal radiotherapy and GLS. However the results of our analysis were not substantively different when these patients were excluded. While radiation increases the cardiotoxicity of anthracyclines via different mechanisms of injury, it is unclear whether this relationship is additive or synergistic.

Overall, 24 of 134 patients (18%) in the current study had abnormal GLS despite a normal LVEF. This is consistent with findings from the St. Jude Lifetime Cohort Study, in which abnormal GLS was more prevalent than a reduction in LVEF among adult survivors of childhood cancer and was associated with both chest-directed radiotherapy and anthracycline exposure [37]. Although GLS has been identified as a robust parameter for early detection and prediction of cardiotoxicity during cancer therapy, the prognostic significance of abnormal GLS among survivors after completion of cancer therapy remains unknown [38]. A recent expert consensus statement from the American Society of Echocardiography highlights the utility of 2DSTE to detect early subclinical LV systolic dysfunction related to cancer treatment, which may allow for better cardiac risk stratification and facilitate timely intervention [24]. Furthermore, the International Late Effects of Childhood Cancer Guideline Harmonization Group recently performed a comprehensive review of the evidence for cardiomyopathy surveillance in survivors of childhood cancer and concluded that echocardiography-based imaging is the preferred modality for surveillance among survivors treated with cardiotoxic therapies [39]. Additional studies are needed to evaluate if early detection of subclinical LV systolic dysfunction using 2DSTE can lead to improved cardiovascular outcomes among cancer survivors.

The findings of this study reinforce the importance of continued routine cardiac surveillance for adult survivors of childhood, adolescent, and young adult cancer many years beyond their initial treatment. Consistent with prior reports, LV systolic dysfunction was detected 15 years after successful treatment in this study, despite only modest doses of anthracycline (median 300 mg/m^2^) exposure. Lipshultz et al. also demonstrated that parameters of LV function progressively declined as many as 15 years after diagnosis among childhood cancer survivors treated with doxorubicin [11]. Given the high prevalence of subclinical LV systolic dysfunction in our study and the potential associated late cardiovascular consequences, continued periodic cardiac surveillance among adult cancer survivors should be an essential component of their long-term care.

This study has several limitations. The prognostic significance of abnormal GLS was not evaluated, and long-term follow-up is currently underway to determine whether abnormal GLS is predictive of subsequent LVEF decline or symptomatic heart failure in this population. Given the limitations of a retrospective cross-sectional study, we assessed LV function at one point in time and cannot comment on longitudinal changes that may have occurred. An analysis of serial echocardiograms is currently underway to investigate the natural history of GLS and LVEF impairment in this at-risk patient population. Although the study did not include a healthy control group, we used reference normative strain data that have been published in several healthy cohorts. Blood pressures were not measured at the time of the echocardiogram which can cause fluctuation in the GLS, but the majority of the patients were normotensive. Finally, our study pools together a heterogeneous study population with multiple cancer types from a single cancer referral center, which may limit the generalizability of the results.

LV systolic dysfunction is significantly more prevalent when assessed by GLS than by 2D LVEF among adult survivors of childhood, adolescent, and young adult cancer. 2DSTE may be useful for the long-term follow-up in this high risk population to identify patients with subclinical LV systolic dysfunction despite a normal LVEF. Mediastinal radiotherapy appears to be an independent risk factor for the development of LV systolic dysfunction in patients treated with anthracycline chemotherapy, and additional studies are needed to further explore the effect of possible confounders. Whether early identification of subclinical LV systolic dysfunction using 2DSTE will translate into long-term cardiovascular benefits warrants further investigation.

## Figures and Tables

**Table 1:** Demographics and treatment characteristics (*n* = 134)^*∗*^.

Characteristic	No.	%	Mean	SD
Current age, years			31.4	8.8
Median	30.5			
Range	18.1–62.6			
Age at diagnosis, years			16.0	9.0
Median	15.8			
Range	0–48.7			
Interval since diagnosis, years			15.4	9.4
Median	15			
Range	2.4–39.6			
Sex				
Male	73	54.5		
Female	61	45.5		
Race				
Non-Hispanic white	114	85.1		
Black	9	6.7		
Other	11	8.2		
Diagnosis				
Sarcoma	54	40.2		
Hodgkin lymphoma	29	21.6		
Acute lymphoblastic leukemia	17	12.7		
Acute myeloid leukemia	14	10.4		
Non-Hodgkin lymphoma	9	6.7		
Other^†^	11	8.2		
Anthracycline cumulative dose exposure			320	124
Median	300			
Range	27–660			
>350	57	42.5		
150–350	58	43.3		
1–150	19	14.2		
Mediastinal RT dose, Gy				
None	82	61.2		
1–30	38	28.4		
>30	14	10.4		
Cardiovascular risk factors				
Hypertension	12	9.0		
Diabetes	7	5.2		
Dyslipidemia	42	31.3		
Treatment with beta-blockers or ACE-I	14	10.4		
Body mass index			24.8	4.7
Median	23.9			
Range	18.0–47.0			
Overweight (BMI 25–<30)	39	29.1		
Obese (BMI ≥ 30)	17	12.7		

^*∗*^RT, radiotherapy; ACE-I, angiotensin converting enzyme inhibitor; BMI, body mass index; SD, standard deviation.

^†^Other: neuroblastoma (*n* = 3), chronic myeloid leukemia (*n* = 2), teratoma (*n* = 2), ependymoma (*n* = 1), nasopharyngeal carcinoma (*n* = 1), retinoblastoma (*n* = 1), and Wilms' tumor (*n* = 1).

**Table 2:** LV size and function by receipt of mediastinal radiotherapy^*∗*^.

	Total (overall)	Mediastinal radiotherapy		*p* value^†^
		Yes (*n* = 52)	No (*n* = 82)	
Fractional shortening, %	33.3 (30.4, 36.1)	33.3 (30.6, 36.0)	33.1 (29.9, 36.1)	0.948
Fractional shortening < 27%	7 (5.2)	5 (9.6)	2 (2.4)	NA
Ejection fraction, %	61.1 (58.0, 63.6)	60.7 (57.6, 63.4)	61.2 (58.1, 64.0)	0.457
Ejection fraction < 55%	8 (6.0)	5 (9.6)	3 (3.7)	NA
GLS, %	18.0 (17.0, 20.0)	18.0 (16.0, 19.5)	19.0 (17.0, 20.0)	0.040
GLS ≤ 16%	31 (23.1)	19 (36.5)	12 (14.6)	0.036
GLS (APLAX), %	18.0 (16.0, 20.0)	17.0 (15.0, 19.0)	19.0 (17.0, 21.0)	0.023
GLS (A4C), %	18.0 (16.0, 20.0)	17.0 (15.0, 19.0)	18.0 (17.0, 20.0)	0.010
GLS (A2C), %	19.0 (17.0, 21.0)	19.0 (16.5, 20.0)	19.0 (17.0, 21.0)	0.279
GLS rate, 1/s	1.1 (1.0, 1.2)	1.1 (1.0, 1.2)	1.1 (1.0, 1.2)	0.995
GRS, %^*∗∗*^	42.1 (31.1, 53.7)	42.1 (26.5, 55.2)	42.0 (31.9, 51.4)	0.843
GRS rate, 1/s^*∗∗*^	2.2 (1.9, 2.7)	2.3 (1.9, 2.9)	2.2 (1.9, 2.7)	0.086
GCS, %^*∗∗*^	17.3 (15.2, 19.7)	16.1 (14.5, 19.7)	17.6 (16.0, 19.7)	0.086
GCS rate, 1/s^*∗∗*^	1.5 (1.3, 1.8)	1.5 (1.3, 1.9)	1.4 (1.3, 1.8)	0.058
LV mass/BSA, g/m^2^	64.3 (55.9, 72.1)	63.1 (56.9, 69.2)	65.4 (55.6, 74.3)	0.099
Mitral *E*/*A* ratio	1.5 (1.2, 1.8)	1.3 (1.0, 1.6)	1.6 (1.3, 1.9)	0.005
Septal *e*′, cm/s	10.4 (8.8, 12.3)	9.3 (8.3, 11.2)	11.6 (9.8, 12.6)	0.003

^*∗*^Numbers are median (interquartile range) for continuous variables and *N* (%) for categorical variables.

GLS, global longitudinal strain; APLAX, apical long axis; A4C, apical 4-chamber; A2C, apical 2 -hamber; LV, left ventricle; BSA, body surface area.

^†^ *p* value from multivariable linear or logistic regression adjusted for continuous cumulative anthracycline dose, sex, and age at echocardiogram.

^*∗∗*^Based on only *n* = 130 patients.
